# A Potential Case of Tetanus-Diphtheria Vaccine-Induced Immune Thrombocytopenia

**DOI:** 10.7759/cureus.101689

**Published:** 2026-01-16

**Authors:** Montserrat Rendon-Beltran, Cesar Mendoza-Maldonado, Carlos A Valdes-Cerda, Jesus A Cabrera-Zambada, Jesus S Velarde-Felix

**Affiliations:** 1 Biomedicine, Faculty of Biology, Universidad Autonoma de Sinaloa, Culiacan, MEX; 2 Biomedical Sciences, Faculty of Chemical and Biological Sciences, Universidad Autonoma de Sinaloa, Culiacan, MEX; 3 Hematology, Servicios de Salud del Instituto Mexicano del Seguro Social para el Bienestar, Hospital General de Culiacan, Culiacan, MEX; 4 Anesthesiology, Hospital General de Ciudad Juarez, Ciudad Juarez, MEX; 5 Genomic Medicine, Servicios de Salud del Instituto Mexicano del Seguro Social para el Bienestar, Hospital General de Culiacan, Culiacan, MEX

**Keywords:** immune thrombocytopenia, platelets, post-vaccination, tetanus-diphtheria vaccine, vaccine-induced itp

## Abstract

Immune thrombocytopenia (ITP) is an autoimmune disorder characterized by autoantibody-mediated platelet destruction. Vaccine-induced ITP, particularly the tetanus-diphtheria (Td) booster, is a well-documented phenomenon in children yet rare among adults. This report describes the case of a 48-year-old woman who presented with petechiae, palate and lingual bleeding, and gastrointestinal hemorrhage one week following Td booster vaccination, along with thrombocytopenia and leukocytosis. Except for uterine fibroids, she had an unremarkable medical history. Serological tests for dengue, HIV, hepatitis B, and hepatitis C, as well as a direct Coombs test, were performed to rule out alternative etiologies of ITP. An integral clinical approach and treatment with steroids and immunosuppressors, following three to four weeks, achieved clinical improvement of ITP. Previous cases and current analysis of the literature suggest molecular mechanisms and genetic factors as probable causes for this outcome; however, detailed clinical history together with environmental and clinical settings may allow for better characterization of this phenomenon.

## Introduction

Immune thrombocytopenia (ITP) is an autoimmune hematologic disorder characterized by an autoantibody-mediated thrombocyte destruction, wherein antibodies target platelet antigens [1]. Although ITP patients develop thrombocytopenia (<100,000 platelets/mm^3^ for ITP diagnosis), the clinical spectrum ranges from asymptomatic to moderate or life-threatening hemorrhage resulting from the depletion of platelets, which are essential for clotting [1,2].

In accordance with international guidelines, ITP is classified into primary and secondary forms based on etiology. Primary ITP occurs in the absence of any apparent cause, whereas the secondary form is induced by concurrent medical conditions or treatments, including autoimmune disorders, malignancies, infectious agents, drugs, and, in extremely rare cases, vaccines, predominantly affecting children. Its diagnosis is based merely on laboratory and clinical features and careful exclusion of all other potential causes [2,3]. It is hypothesized that vaccines may trigger autoimmunity via molecular mimicry and epitope spreading, leading to thrombocytopenia. The appearance of severe ITP after vaccination has been documented across a variety of vaccines, being COVID-19 the most recent and studied example [4]. In this case report, we present a potentially vaccine-induced ITP following tetanus-diphtheria (Td) vaccine administration in a healthy adult woman, highlighting the difficulties encountered in the diagnosis and treatment during the course of the disorder.

## Case presentation

A 48-year-old woman arrived at the emergency room (ER) after sustaining a traumatic injury on her right third finger due to an accidental entrapment in a residential door, in search of medical attention to stop the bleeding, clean the wound to prevent infection, and undergo a surgical procedure to recover. The mechanism of injury involved significant crushing force, resulting in an ungual avulsion with amputation of the distal phalanx, with extensive epithelial and connective tissue loss involving osseous structures. Following the trauma, the physician prescribed an analgesic and antibiotic 10-day regimen, including dexketoprofen (25 mg every 8 hours) and paracetamol/tramadol (325/37.5 mg every 12 hours), alongside cephalexin (500 mg every 8 hours).

A day after the incident, a single dose of Td vaccine (2805X003A, Serum Institute of India, Pune, India) was administered. Seven days later, she noticed the abrupt appearance of petechiae in lower and upper extremities and a sensation of tachycardia, without other bleeding incidents.

She was an apparently healthy individual, denying allergies, arterial hypertension, and diabetes mellitus. She reported passive exposure to tobacco smoke and denied alcohol consumption. Her medical history is positive for uterine fibroids, and she did not remember a previous vaccination for tetanus or diphtheria. Currently, she possesses an intrauterine device (IUD) for contraception.

Upon admission to the ER, the physical examination revealed normal neurological functions, afebrile (36.6°C), a respiratory rate of 20 breaths per minute, without organomegaly, with bowel sounds being present and normal, but with tachycardia (124 beats per minute), and hypertension (blood pressure: 160/100 mmHg).

Laboratory findings revealed severe thrombocytopenia (9,000 platelets/mm^3^; reference range: 150,000-450,000/mm^3^), leukocytosis (11.02 × 10^3^/µL; reference range: 5.00-10.00 × 10^3^/µL), normal hemoglobin values (13.8 g/dL; reference range: 12.00-18.00 g/dL), as well as an elevated erythrocyte sedimentation rate (Table 1).

**Table 1:** Monitoring of hematological parameters during patient’s evolution

Tet	Admission	Day 3	Day 8	Day 20	Day 24	Follow-up	Reference range
White blood cells	11.02	7.42	16.03	14.59	20.43	13.00	5.00-10.00 × 10^3^/µL
Platelet count	9,000	6,000	53,000	17,000	153,000	215,000	150,000-450,000/mm^3^
Hemoglobin	13.8	11.8	13.4	14.5	13.9	13.20	12.00-18.00 g/dL
Hematocrit	42.3	35.4	39.3	44.6	41.9	40.5	37.00-51.00%
Mean cell volume	89.8	88.7	88.2	92.5	92.4	94.30	80.00-97.00 fL
Mean cell hemoglobin	29.3	29.5	30.0	30.1	30.6	30.9	26.00-32.00 pg
Mean platelet volume	-	-	12.6	8.1	11.1	10.20	9.70-10.90 fL
Prothrombin time	13.1	-	-	-	-	-	10-16 s
Partial thromboplastin time	32.0	-	-	-	-	-	25-45 s

Within routine blood chemistry analysis, the fasting plasma glucose, alanine aminotransferase, and aspartate aminotransferase values were abnormal (Table 2). Serological tests for HIV, hepatitis B, and hepatitis C, as well as a direct Coombs test, were negative. An Enzyme-Linked Immunosorbent Assay (ELISA) test revealed high levels of anti-toxoid tetanic antibodies (5.33 IU/mL; reference range: 0.00-0.10 IU/mL). By appearance of petechiae, a serological test for dengue was performed, resulting in a positive test for IgM/IgG. However, due to the absence of common symptoms of dengue (fever, headache, myalgia, arthralgia, and nausea), reverse transcription polymerase chain reaction (RT-PCR) and NS1 for dengue virus were performed, both resulting in negative results, discarding acute dengue infection, leading to the final diagnosis of ITP.

**Table 2:** Biochemical profile and additional laboratory data obtained upon patient’s admission BUN: blood urea nitrogen; LDH: lactate dehydrogenase; ALT: alanine transaminase; AST: aspartate transaminase; ALP: alkaline phosphatase; ESR: erythrocyte sedimentation rate

Test	Result	Reference range
Glucose	139.49	70.00-105.00 mg/dL
Urea	30.88	10.00-55.00 mg/dL
Creatinine	0.65	0.60-1.10 mg/dL
BUN	14.43	7.00-18.00 mg/dL
Uric acid	3.66	2.00-6.00 mg/dL
Sodium	143	135-148 mmol/L
Potassium	3.7	3.5-5.3 mmol/L
Chloride	104	95.00-110.00 mmol/L
Phosphorus	2.14	2.50-4.90 mg/dL
Magnesium	1.97	1.80-2.40 mg/dL
Calcium	9.93	8.50-10.10 mg/dL
LDH	343.42	240-480 UI/L
Total bilirrubin	0.33	0.00-1.00 mg/dL
ALT	18.10	21.00-55.00 U/L
AST	12.39	17.00-59.00 U/L
ALP	151.52	53.00-198.00 U/L
Albumin	4.49	3.50-5.00 g/L
ESR	18	0-15 mm/Hr

Chronologically, the hematological treatment started with dexamethasone (40 mg) for three days. After that, on the second day of hospitalization, she developed an acute but slight decrease in hemoglobin value (11.8 g/dL), and both platelet count (6,000 platelets/mm^3^) and hematocrit (35.4%) levels dropped, compared to baseline. For this reason, the patient received two platelet transfusions, obtained by apheresis, in addition to starting a 20-day treatment regimen consisting of azathioprine (50 mg) every 24 hours and prednisone, structured as a tapering dose every five days (100 mg, 75 mg, 50 mg, and 12.5 mg, respectively).

On day 2, the patient exhibited palatal and tongue bleeding, as well as melena. Moreover, a peripheral blood smear was performed, observing a low percentage of platelets along with the absence of blasts.

By day 6, the oral mucosal lesions and melena had resolved. In the follow-up laboratory tests on day 8, the thrombocytopenia persisted (53,000 platelets/mm^3^), but the patient was discharged due to clinical improvement, with instructions to return immediately upon any sign of bleeding.

**Figure 1:**
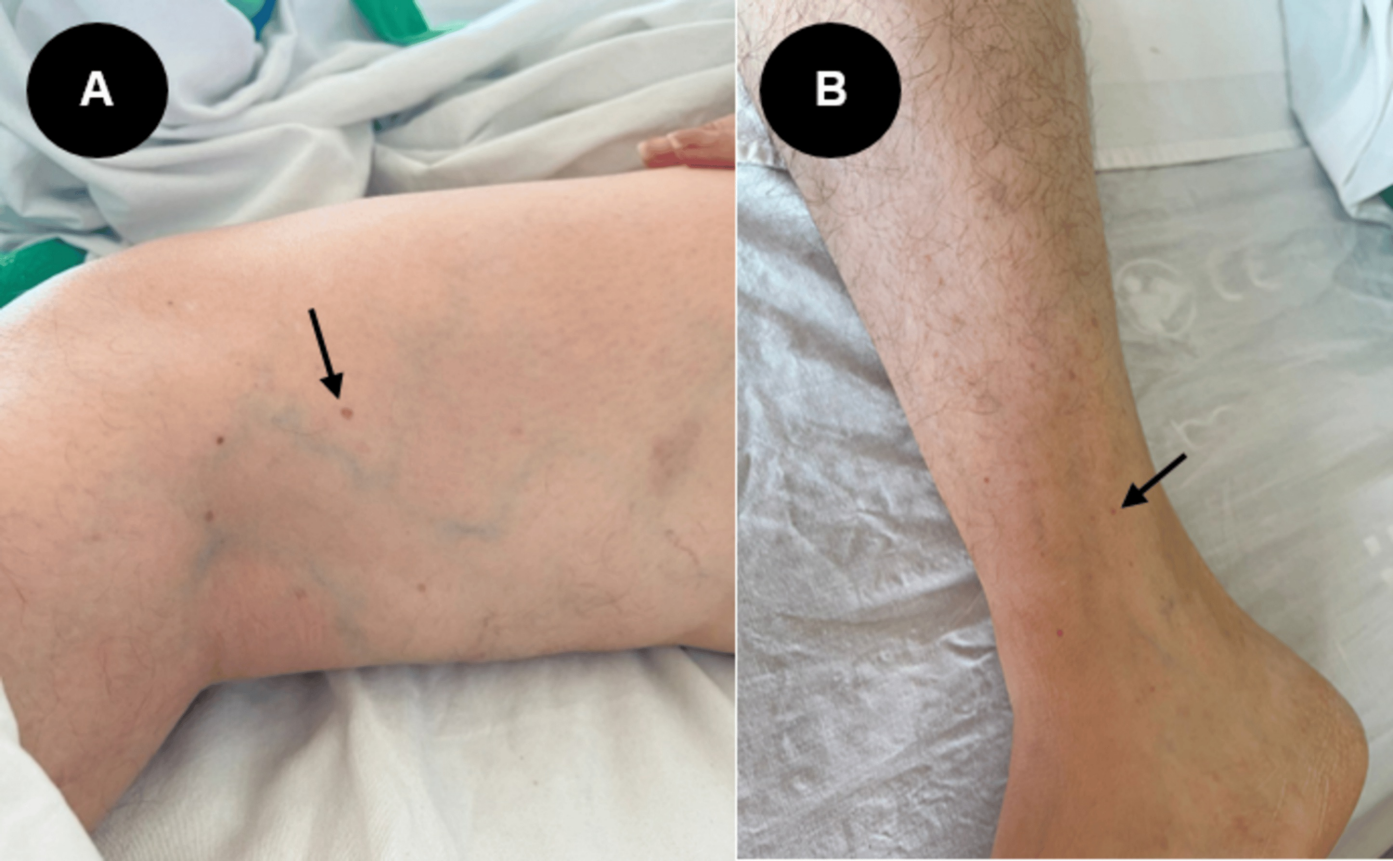
Petechiae observed on the patient's lower limbs during readmission Hamstring (A) and ankle (B) petechiae, as indicated by the arrows

Twelve days later, the patient returned to the hospital due to the presence of blood in stool and persistent petechiae on the extremities (Figure 1). Laboratory tests demonstrated severe thrombocytopenia (17,000 platelets/mm^3^); therefore, she was readmitted to the hospital.

**Figure 2:**
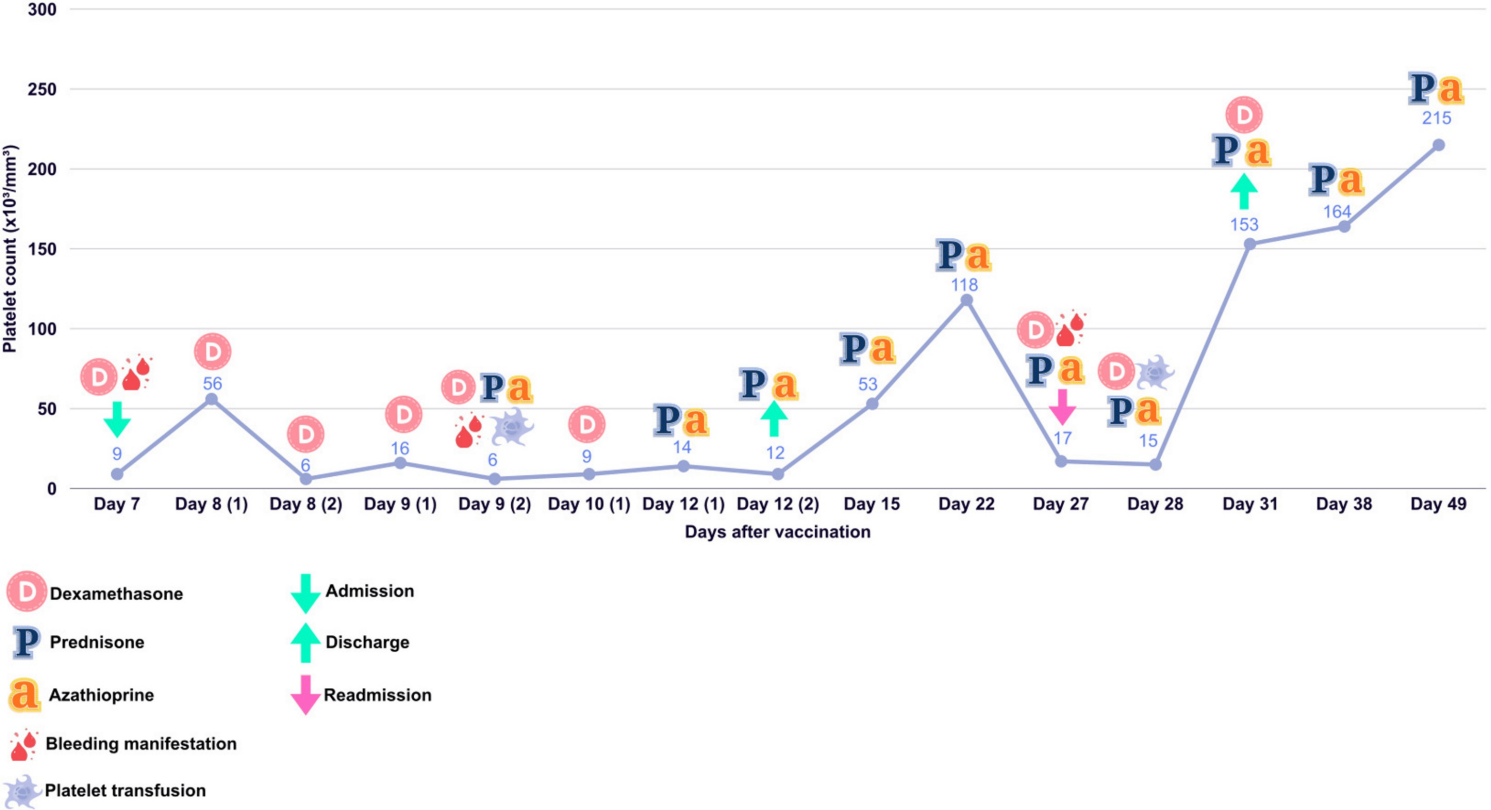
Platelet count progression: a timeline

Her second admission lasted four days. During this period, the hematologist prescribed dexamethasone (40 mg) every 24 hours for four days, maintaining the azathioprine and prednisone regimen, and an additional platelet transfusion was administered. The patient was monitored with regular blood counts, showing a progressive increase in platelet levels. After completing treatment with dexamethasone and in the absence of further complications, the patient was discharged. Three weeks later, at a follow-up appointment, the patient exhibited a normal platelet count (Figure 2) and remained asymptomatic.

## Discussion

In this case report, we present the onset of thrombocytopenia in an adult woman following the administration of a Td booster. The patient was hospitalized due to the appearance of petechiae on the extremities and severe thrombocytopenia (9,000 platelets/mm³). During the initial admission, she exhibited hemorrhagic manifestations, including palate and lingual bleeding, as well as melena.

Twelve days after discharge, the patient required readmission following the recurrence of blood in stool. Throughout her clinical course, she received three platelet transfusions and was managed with a therapeutic regimen comprising dexamethasone, prednisone, and azathioprine.

Despite the patient's history of uterine fibroids, she presents no other chronic conditions that could compromise her immunological status. In search of the precise etiology of ITP, various diagnostic tests were performed, among them HIV, hepatitis B, hepatitis C, and NS1 for dengue, which were negative, but positive for IgM/IgG for dengue. However, dengue infection was discarded with a negative result in an RT-PCR for Zika, chikungunya, and dengue. This finding was interpreted as a false positive, consistent with prior studies indicating that false-positive results may arise from prior flavivirus infections or underlying autoimmune disorders [5,6], such as systemic lupus erythematosus (SLE) [7] or, as in this case, ITP.

Literature regarding the association between the Td vaccine and ITP is limited; to date, only two cases involving adults have been reported. Serinken et al. [8] published the case of a 48-year-old male patient with an unremarkable medical history who presented with hematuria and purpura on the extremities three days after receiving a second dose of the bivalent Td vaccine, exhibiting a platelet count of 4,200/mm³ upon admission. Similar to our case, he was treated with high-dose corticosteroids and achieved a normal platelet count after one month of treatment. In Belgium, Mortelmans et al. [9] reported the case of a 40-year-old woman who developed epistaxis, subconjunctival hemorrhage, purpura, and ecchymosis four days following the vaccine booster, reaching a nadir of 1,000 platelets/mm³. Most ITP cases associated with vaccines containing Td toxoids have been reported with combined formulations, such as diphtheria, pertussis, and tetanus (DPT)-oral polio vaccine (OPV); diphtheria, tetanus, acellular pertussis (DTaP)-inactivated poliovirus (IPV); and DPT-*Haemophilus influenzae *type b (HIb) [2,10,11].

Although vaccines play a critical role in the prevention and control of infectious diseases, adverse events following immunization have been reported, including pain, swelling, erythema, fever, and rash at the injection site [12]. The development of ITP after vaccination has been primarily reported in association with the MMR vaccine in the pediatric population, whether administered alone or in combination with other agents [13-15]. In 2011, Okazaki et al. [16] detected anti-measles and anti-rubella IgG antibodies on the platelet surface of an 18-month-old female patient, three weeks after the administration of the measles-rubella (MR) vaccine alongside varicella and mumps vaccines. This finding is crucial for understanding that vaccine-induced ITP involves a distinct immunological component. Other vaccines implicated in ITP include influenza [17], hepatitis B [18], and herpes zoster [19]. More recently, Pfizer and Moderna SARS-CoV-2 vaccines [20,21] have been associated with the condition, as well as cases of vaccine-induced thrombotic thrombocytopenia (VITT), triggered by the Oxford-AstraZeneca vaccine [22,23]. Proposed molecular mechanisms underlying vaccine-induced autoimmunity include molecular mimicry, epitope spreading, and polyclonal activation [24]. The mechanism underlying the thrombotic thrombocytopenia caused by COVID-19 adenoviral vaccines is well studied and is categorized as a form of anti-PF4 syndrome [25]. In contrast, patients with ITP do not present with clotting episodes, suggesting a different pathway, which remains unknown. The loss of immune tolerance involves both genetic and immune factors, as demonstrated by Petito et al. [26], who identified three HLA class II polymorphisms (-DPB117:01, -DQA105:01, and -DRB1*11:04), significantly associated with VITT in the Italian population. Also, Amin et al. described various HLA alleles associated with ITP, thrombotic thrombocytopenia purpura (TTP), and heparin-induced thrombocytopenia (HIT) [27]. Unfortunately, in the present case, this analysis was not possible to perform.

## Conclusions

This case reports the onset of ITP in an adult woman following the administration of a Td booster, highlighting the difficulties encountered during diagnosis and treatment. Although a direct causality between the Td vaccine and ITP cannot be definitively established due to the scarcity of case reports and clear evidence, the rapid development of severe hemorrhagic manifestations suggests a potential association. Since the bivalent Td vaccine is generally administered as a booster for adolescents and adults every 10 years, and its use is also recommended following injuries with a risk of infection, clinicians should be aware that this represents a very rare but potentially life-threatening adverse reaction.
